# Dynamic Transcriptomic and Cellular Remodeling Underlie Cuprizone-Induced Demyelination and Endogenous Repair in the CNS

**DOI:** 10.3390/antiox14060692

**Published:** 2025-06-06

**Authors:** Yantuanjin Ma, Tianyi Liu, Zhipeng Li, Wei Wei, Qiting Zhao, Shufen Wang

**Affiliations:** 1 Yunnan Key Laboratory of Breast Cancer Precision Medicine, Institute of Biomedical Engineering, Kunming Medical University, Kunming 650500, China; 20231857@kmmu.edu.cn (Y.M.);; 2 College of Basic Medical Sciences, Kunming Medical University, Kunming 650500, China

**Keywords:** cuprizone, demyelination, remyelination, blood–brain barrier integrity, glial cell, neuroinflammation, snRNA-seq, RNA-seq

## Abstract

Demyelination in the central nervous system (CNS) disrupts neuronal communication and promotes neurodegeneration. Despite the widespread use of cuprizone-induced demyelination models to study myelin injury and repair, the mechanisms underlying oligodendrocyte apoptosis and regeneration are poorly understood. This study investigated the dynamic cellular and molecular changes that occur during demyelination and remyelination, with a focus on glial cell responses, blood-brain barrier (BBB) integrity, and neuroimmune interactions. *C57BL/6J* mice exposed to cuprizone exhibited weight loss, sensorimotor deficits, and cognitive decline, which were reversed during remyelination. Histological and immunofluorescence analyses revealed reduced myelin protein levels, including myelin basic protein (MBP) and myelin-associated glycoprotein (MAG), and decreased oligodendrocyte populations during demyelination, with recovery during repair. The BBB permeability increases during demyelination, is associated with the decreased expression of tight junction proteins (ZO-1, Occludin), and normalizes during remyelination. Single-cell RNA sequencing revealed dynamic shifts in glial cell populations and upregulated Psap-Gpr37l1 signaling. Neuroimmune activation and oxidative stress peak during demyelination, characterized by elevated ROS, MDA, and immune cell infiltration, followed by recovery. Transcriptomic profiling revealed key inflammatory pathways (JAK-STAT, NF-κB) and hub genes associated with demyelination and repair. These findings provide insights into myelin repair mechanisms and highlight potential therapeutic targets for treating demyelinating diseases.

## 1. Introduction

Myelin within the central nervous system is a structurally intricate, multilayered membrane that tightly ensheathes nerve fibers, providing electrical insulation and support to ensure the efficient propagation of nerve impulses [1]. Primarily synthesized by oligodendrocytes, myelin is rich in lipids and specific proteins and is distributed across various regions of the brain and spinal cord [2,3]. When the myelin integrity is compromised, the resulting loss of axonal insulation not only impedes nerve impulse transmission but also triggers irreversible neurodegenerative processes, forming the core pathological mechanism in neurological diseases such as multiple sclerosis [4,5]. Although hereditary disorders of myelin development are relatively rare [6], the interplay of environmental pathogens [7], metabolic dysregulation [8], and immune dysfunction [9] constitutes the major contributing factor to the more prevalent acquired demyelinating lesions observed in clinical practice.

Cuprizone is commonly employed in experimental models to induce demyelination by disrupting the mitochondrial function within oligodendrocytes, leading to energy metabolism imbalance and the activation of apoptotic pathways [10,11]. The apoptosis of oligodendrocytes results in unavoidable myelin degradation, recapitulating clinical demyelinating disease features [12,13]. Upon the cessation of cuprizone administration, endogenous repair mechanisms are activated, triggering remyelination, thus providing an ideal model for studying injury and repair processes [14]. However, traditional feeding methods are limited in their precise experimental designs because of significant fluctuations in intake and potential systemic toxic side effects [15,16]. The adoption of intragastric administration methods enables accurate dose titration and improved reproducibility, thereby advancing the study of the critical molecular mechanisms underlying demyelination and remyelination.

Glial cells play pivotal roles in regulating injury responses and repair processes following CNS damage [17]. Microglia are swiftly activated to phagocytose myelin debris [18], whereas reactive astrocytes proliferate and secrete a range of cytokines and growth factors, creating a multifaceted inflammatory milieu [19]. Recent studies have revealed profound metabolic reprogramming in glial cells under stress conditions, and that microglia shift toward glycolysis to meet phagocytosis energetic demands [20], whereas astrocytes restructure lipid metabolism to mitigate oxidative stress [21]. This metabolic reprogramming may critically influence the outcome of myelin regeneration. Furthermore, fibrinogen deposition resulting from BBB leakage has been identified as a factor that amplifies inflammation through the activation of innate immune receptors, potentially establishing a detrimental feedback loop that hinders repair processes [21,22].

In this study, CPZ was administered via gavage to achieve precise dosing in demyelination and remyelination experimental models and to assess the BBB permeability in treated mice. By integrating histological examination with RNA sequencing, a comprehensive analysis of the transcriptomic characteristics across various stages of demyelination and remyelination was conducted, aiming to elucidate the key molecular mechanisms underlying oligodendrocyte apoptosis and regeneration.

## 2. Methods

### 2.1. Mice and Experimental Model

**Figure 1:**
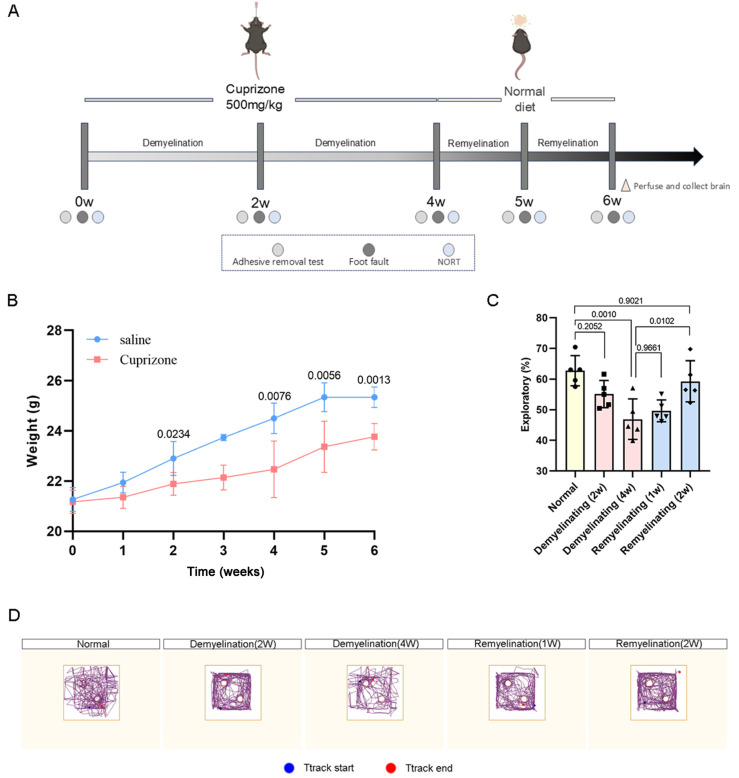
Alterations in sensorimotor and cognitive functions across demyelination and remyelination in cuprizone-induced mouse model. (**A**) Experimental timeline of cuprizone-induced mouse model. (**B**) Body weight measurements of cuprizone-administered mice at various time points. (**C**) Evaluation of short-term memory changes during demyelination and remyelination via novel object recognition test (NOR). (**D**) Behavioral trajectory plots. All *p* values were calculated via one-way ANOVA, followed by Tukey’s multiple comparison test.

Male *C57BL/6J* mice, aged 10 weeks, were procured from the Animal Medical Department of Kunming Medical University (SYXK(DIAN)K2020-006). The mice were housed under specific pathogen-free (SPF) conditions with a 12 h light/dark cycle at a density of four per cage. All experimental procedures received approval from the Animal Ethics Committee of Kunming Medical University (Protocol Number: kmmu20230296). Demyelination was induced by daily oral gavage of 0.5% cuprizone (500 mg/kg) suspended in corn oil for four consecutive weeks. Control mice received the same volume of corn oil on the same schedule. Following the 4-week treatment period, the cuprizone administration ceased to allow spontaneous remyelination. Throughout the experiment, all the animals had ad libitum food and water. Mice from the control, demyelination, and remyelination groups were sacrificed concurrently (Figure 1A).

### 2.2. Neurobehavioral Tests

Behavioral assessments were conducted to evaluate neurological function changes in the mice during the cuprizone-induced demyelination and remyelination phases. Sensorimotor deficits were assessed via the tape removal test and foot fault test following established protocols [23]. A 4 × 3 mm adhesive tape was applied to the hairless region of the forepaw, and the time taken for the mouse to touch and remove the tape was recorded. If the mouse failed to touch or remove the tape within 120 s, the time was recorded as 120 s. The foot fault test was used to evaluate sensorimotor coordination during spontaneous movement. The mice were placed on an elevated grid 30 cm above the ground and were allowed to walk freely for 1 min. The total number of steps and foot faults was recorded.

The novel object recognition (NOR) test was employed to assess the learning and memory in the rodents [24]. The mice were tested via a two-phase procedure: (1) Training phase: Mice freely explored two identical objects in a square open field for 10 min, followed by a 10 min rest period. (2) Testing phase: One object was replaced with a novel object, and the mice explored both objects for 10 min. The preference index was calculated as the ratio of the time spent exploring the novel object to the total exploration time [25].

### 2.3. Luxol Fast Blue Staining

For the histopathological evaluation, deep anesthesia was induced via sodium pentobarbital (0.3%, 40 mg/kg), followed by transcardial perfusion in PBS. The brain tissues were carefully harvested and fixed in 4% paraformaldehyde before being embedded in paraffin. Serial 5 μm thick sections were prepared for subsequent staining. The degree of demyelination was assessed via Luxol Fast Blue (LFB) (L0294, Sigma-Aldrich, St. Louis, MO, USA) staining. The stained sections were examined under a light microscope (Nikon ECLIPSE 80i, Nikon Corporation, Tokyo, Japan), and digital images were captured for further analysis.

### 2.4. Evans Blue Permeability Assay

To evaluate the BBB integrity, a 2% Evans blue (EB) solution was injected retro-orbitally and was allowed to circulate for 2 h. Deep anesthesia was then induced via sodium pentobarbital, followed by transcardial perfusion with PBS for brain tissue extraction. Whole-brain EB infiltration was documented via a high-resolution digital imaging system. For quantitative analysis, brain tissues were homogenized in 500 μL of 50% (*w*/*v*) trichloroacetic acid via an ultrasonic homogenizer (HD4400, WIGGENS, Straubenhardt, Germany) in an ice bath. The homogenate was incubated at 4 °C for 24 h and centrifuged at 13,000× *g* for 30 min. The supernatant was collected, and the absorbance at 620 nm was measured via a multimode microplate reader (Infinite M200 PRO, Tecan, Tecan, Männedorf, Switzerland). The concentration of extravasated dye was determined via a standard curve and expressed as μg/g of brain tissue.

### 2.5. FITC–Dextran Tracer Assay

A 200 μL solution of FITC-labeled dextran (10 kDa, 1 mg/mouse) was administered via retro-orbital venous plexus injection, allowing systemic circulation for 2 h. The brain tissues were carefully extracted without transcardial perfusion and were subsequently fixed in 4% paraformaldehyde. The samples were sequentially dehydrated in a 30% (*w*/*v*) sucrose solution, embedded in OCT compound, and cryosectioned at −20 °C. Coronal sections (30 μm) were prepared via a cryostat and mounted onto slides pretreated with 0.05% (*w*/*v*) poly-L-lysine. The sections were sealed with an antifade mounting medium to preserve the fluorescence signals. Imaging was performed via a laser scanning confocal microscope (LSM880, Zeiss, Oberkochen, Germany), and the fluorescence intensity was quantified via the ZEN 3.0 imaging analysis system.

### 2.6. Immunofluorescence Staining

Following transcardial perfusion in ice-cold PBS, brain tissues were carefully extracted and fixed in 4% paraformaldehyde (PFA, pH 7.4) for 24 h. The samples were cryoprotected in 30% (*w*/*v*) sucrose, embedded at the optimal cutting temperature (OCT), and frozen at −20 °C. Coronal sections (20 μm) were prepared via a cryostat. After being washed in PBS, the sections were permeabilized and blocked for 2 h at room temperature in PBS containing 5% donkey serum and 0.3% Triton X-100. Primary antibody incubation was performed overnight at 4 °C in PBS supplemented with 5% donkey serum and 0.3% Triton X-100. The primary antibodies used included anti-MAG (1:300, AB277535, Abcam, Cambridge, UK), anti-MBP (1:500, AB7349, Abcam, Cambridge, UK), anti-PDGFRα (1:100, AF1062, R & D Systems, Minneapolis, MN, USA), anti-CC1 (1:500, AB239828, Abcam), anti-Olig2 (1:200, PRB009, Oasis Biofarm, Hangzhou, China), anti-ZO-1 (1:100, AB307799, Abcam), anti-Occludin (1:100, 80545, Proteintech, Wuhan, China), anti-GFAP (1:500, AB32760, Abcam), and anti-IBA1 (1:500, AB32760, Abcam) antibodies. After washing, the sections were incubated with secondary antibodies at room temperature for 1 h. The secondary antibodies used included Alexa Fluor 488-conjugated anti-rabbit IgG (1:500, A-21206, Thermo Fisher, Waltham, MA, USA), Alexa Fluor 488-conjugated anti-mouse IgG (1:500, A-28175, Thermo Fisher, MA, USA), Alexa Fluor 594-conjugated anti-rabbit IgG (1:500, A-11037, Thermo Fisher, MA, USA), Alexa Fluor 594-conjugated anti-mouse IgG (1:500, A-11032, Thermo Fisher, MA, USA), and Alexa Fluor 594-conjugated anti-rat IgG (1:500, A-21208, Thermo Fisher, MA, USA). The sections were counterstained and mounted onto a DAPI-containing antifade mounting medium. Fluorescence imaging was performed via a laser scanning confocal microscope (LSM880, Zeiss), and quantitative analysis of the fluorescence intensity was conducted via ZEN 3.0 imaging software.

### 2.7. Flow Cytometry

Brain mononuclear cells were isolated via Percoll density gradient centrifugation (17-0891, GE Healthcare, Chicago, IL, USA). Following Fc receptor blockade, the cell suspensions were incubated with fluorescence-conjugated monoclonal antibodies at 4 °C for 30 min in the dark. The antibodies used included anti-mouse CD45-PerCP (103130, BioLegend, San Diego, CA, USA), anti-CD3-FITC (100204, BioLegend, CA, USA), and anti-F4/80-APC (123115, BioLegend, CA, USA). After staining, the cells were washed twice in PBS containing 2% fetal bovine serum (FBS) and were resuspended in 300 μL of PBS. Flow cytometry was performed using a FACSCanto II (BD Biosciences, CA, USA) instrument equipped with 488 nm and 640 nm lasers. The data were analyzed via FlowJo software (version 10.1), and the representative gate strategies are presented in Supplementary Figure S1A.

### 2.8. Western Blot

Total protein was extracted from tissue samples via RIPA lysis buffer (P0013B, Beyotime, Shanghai, China), while membrane proteins were isolated via a dedicated extraction kit (P0033, Beyotime, Shanghai, China), with protease inhibitors added to prevent protein degradation. The protein concentration was quantified via a BCA protein assay kit (P0011, Beyotime, Shanghai, China). Equal amounts of protein samples were subjected to sodium dodecyl sulfate-polyacrylamide gel electrophoresis (SDS-PAGE) and transferred to polyvinylidene fluoride (PVDF) membranes (Millipore, Burlington, MA, USA). The membranes were incubated with primary antibodies, including anti-MAG (1:2000, AB277535, Abcam), anti-MBP (1:2000, AB7349, Abcam), anti-ZO-1 (1:1000, AB307799, Abcam), anti-Occludin (1:1000, 80545, Proteintech, Wuhan, China), anti- NF-κB p65 (1:2000, 8242T, Cell Signaling Technology), and anti- Phospho-NF-κB p65 (1:2000, 3033T, Cell Signaling Technology, Danvers, MA, USA) antibodies. HRP-conjugated secondary antibodies were subsequently applied, and protein bands were visualized via a chemiluminescence imaging system. Band intensities were quantified with ImageJ software, version 1.54.

### 2.9. Quantitative Real-Time PCR (qRT-PCR)

Total RNA was extracted from the cortical and corpus callosum tissues using the TRIzol reagent (15596018, Thermo Fisher Scientific, Waltham, MA, USA). The RNA was then reverse-transcribed into cDNA via the PrimeScript RT reagent kit (TK1621, Thermo Fisher Scientific), with 1 μg of total RNA used as input. Quantitative real-time PCR (qRT-PCR) was performed on an ABI Q6 Real-Time PCR System (Applied Biosystems, Foster City, CA, USA) with SYBR Green Master Mix (Roche) and specific primers (sequences are provided in Supplementary Table S1). The reaction conditions were set as follows: 1 min of predenaturation at 95 °C, followed by 40 cycles of 5 s of denaturation at 95 °C and 30 s of annealing/extension at 60 °C. The gene expression was quantified by the 2-ΔΔCq method.

### 2.10. Oxidative Stress-Related Factor Detection

The brain tissue was minced and filtered through 70 μm mesh to obtain a single-cell suspension. The levels of intracellular reactive oxygen species (ROS) were quantified via a DCFH-DA Flow Cytometry Kit (S0033S, Beyotime, Shanghai, China) via flow cytometry. Brain tissue was homogenized in PBS, and supernatant was collected. The malondialdehyde (MDA) content and superoxide dismutase (SOD) activity were determined via the thiobarbituric acid method (S0131S, Beyotime, Shanghai, China) and the WST-1 assay (A001-3-2, Nanjing Jiancheng Bioengineering Institute, Nanjing, China) kits, respectively, according to the manufacturers’ instructions. The data were normalized to the total protein concentration measured via the BCA method.

### 2.11. scRNA-seq and Data Mining

scRNA-seq count data from the cortex and corpus callosum of normal and cuprizone-induced demyelinated mice were downloaded from the GEO database (GEO accession number: GSE204769). A standard Seurat analysis pipeline was employed to construct Seurat objects, with a more stringent mitochondrial content threshold (5%) applied during data filtering. The data were log-normalized via the normalizeData function, and the mitochondrial gene percentages were regressed during data scaling. Principal component analysis (PCA) identified the top 3000 most variable genes, whereas UMAP dimensionality reduction utilized the first 20 principal components. Clustering analysis was performed with a resolution of 0.3. Manual inspection and detailed annotations were subsequently performed on the basis of widely accepted cell type markers and annotations from the original publication’s authors [26]. Cell-cell communication networks were inferred via the CellChat and CellPhoneDB tools on the basis of single-cell transcriptomics data. Briefly, expression data were loaded, and a CellChat object was created using the Seurat object. Ligand-receptor databases were then loaded and filtered for information related to “secretory signaling,” and overexpressed genes and their interactions in each cell type were identified. Intercellular communication probabilities were calculated, and communication signals involving fewer than 10 cells were excluded. Pathway-level communication networks were then inferred. Differentially expressed genes (DEGs) in subclusters from different experimental groups were visualized via volcano plots. Pseudobulk analysis was applied to the DEG analyses. The thresholds for DEG selection were an absolute log fold change (FC) greater than 0.585 and a *p* value less than 0.05. The resulting gene list was used as input for the subsequent pathway analysis. A Venn diagram was generated via InteractiVenn (http://www.interactivenn.net/ (accessed on 5 October 2024)) to visualize the overlaps and relationships between the DEGs. To further explore the functional significance of the overlapping DEGs, KEGG pathway enrichment analysis was performed via the clusterProfiler package, and significantly enriched pathways (*p* < 0.05) were visualized via a bubble plot.

### 2.12. Bulk Transcriptome Analysis

Total RNA was extracted from cortical and whole-brain tissue samples via TRIzol reagent (15596018, Thermo Fisher Scientific, MA, USA). Sequencing libraries were constructed with the Illumina TruSeq Stranded Total RNA Sample Prep Kit, ensuring that the input RNA quantity met the standard quality control threshold of ≥1 μg. Paired-end 150 bp sequencing was performed on the Illumina NovaSeq 6000 platform. Following quality assessment, the raw sequencing reads were aligned to the *C57BL/6J* mouse reference genome (Ensembl release 100) via HISAT2 (v2.2.1). Differentially expressed genes (DEGs) were identified across groups and samples via the DESeq2 package, with a significance threshold set at |log2FC| > 1 and *p* < 0.05.

To further elucidate the gene coexpression network characteristics, a scale-free topology network was constructed via weighted gene coexpression network analysis (WGCNA). Coexpression modules were identified through a dynamic tree-cut algorithm, and correlations between module eigengenes and phenotypic traits were computed. Gene Ontology (GO) functional annotation and KEGG pathway enrichment analyses were performed on genes within significant modules and DEGs. Overlapping genes from the DEGs and key module genes were imported into the STRING database to construct a protein-protein interaction (PPI) network, applying a confidence threshold of >0.7. The network topology was visualized via Cytoscape, version 3.10.1, and the hub genes were identified via the cytoHubba algorithm (Degree).

### 2.13. Statistical Analysis

All the experiments were performed at least three times, and the data are presented as the means ± standard deviations (SDs). Statistical analysis of the validation experiments was conducted via Prism 8.0 software. One-way analysis of variance (ANOVA) followed by Tukey’s multiple comparison test was applied to assess the statistical significance, with a predefined significance threshold of α = 0.05. For omics data, the Benjamini–Hochberg method was applied for corrections to ensure the stability of the differentially expressed gene identification and pathway enrichment results.

## 3. Results

### 3.1. Neurological and Motor Functions in Cuprizone-Induced Mice

To evaluate the progressive effects of cuprizone-induced demyelination and remyelination on cognitive and sensorimotor functions, behavioral assessments were conducted at multiple time points: control, 2-week and 4-week demyelination, and 1-week and 2-week remyelination (Figure 1A). The mice exhibited significant weight loss during the 4-week demyelination phase, which persisted into the remyelination phase but showed partial recovery (Figure 1B). Sensorimotor deficits emerged from the second week of demyelination, as evidenced by the impaired performance in the adhesive removal and foot fault tests, followed by a marked improvement after 2 weeks of remyelination (Supplementary Figure S1B,C), suggesting partial functional restoration. In the novel object recognition test, mice subjected to 2-week and 4-week demyelination demonstrated reduced interaction with novel objects, indicating cognitive impairment. During remyelination, cognitive deficits were partially ameliorated, as reflected by an increase in the exploration time (Figure 1C,D).

### 3.2. Dynamic Reconstruction of Oligodendrocytes During Cuprizone-Induced Demyelination and Endogenous Remyelination

**Figure 2:**
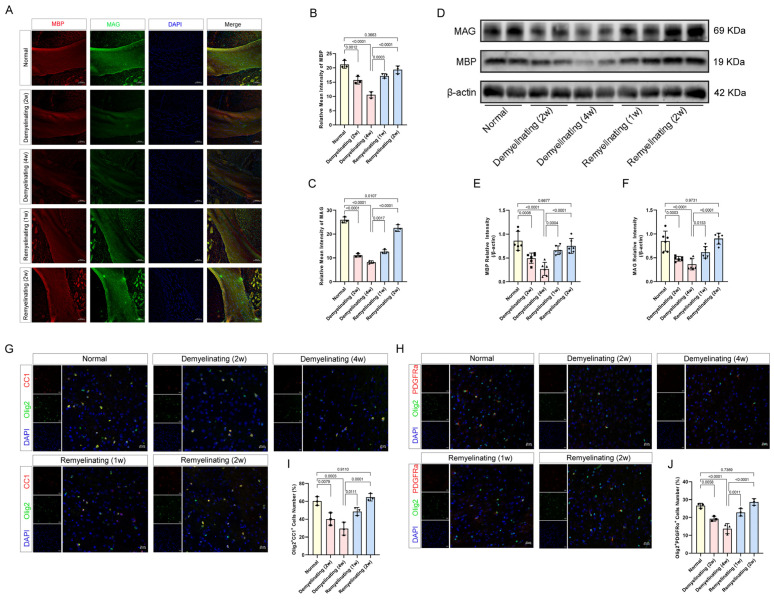
Cuprizone-induced demyelination. (**A**) Representative immunofluorescence images of MBP (red) and MAG (green) in corpus callosum at different time points during demyelination and remyelination (scale bar: 100 µm). (**B**,**C**) Quantitative analysis of MBP and MAG relative intensities. (**D**) Western blot showing changes in MBP and MAG protein expression. (**E**,**F**) Quantitative analysis of the ratios of MBP/β-actin and MAG/β-actin from Western blot images. (**G**) Representative immunofluorescence images of CC1 (red) and Olig2 (green) in the corpus callosum at different time points during demyelination and remyelination (scale bar: 20 µm). (**H**) Representative immunofluorescence images of PDGFRα (red) and Olig2 (green) in the corpus callosum at different time points during demyelination and remyelination (scale bar: 20 µm). (**I**) Normalized percentage of CC1^+^Olig2^+^ cells relative to the total Olig2^+^ population. (**J**) Normalized percentage of PDGFRα^+^Olig2^+^ cells relative to the total Olig2^+^ population. All *p* values were calculated via a one-way ANOVA, followed by Tukey’s multiple comparison test.

The observed deficits in cognitive and sensorimotor functions following cuprizone exposure suggest dynamic regulation by the processes of demyelination and remyelination. In the cuprizone-treated mouse model, the stage-dependent remodeling of corpus callosum myelin was evident. Quantitative Luxol fast blue (LFB) histochemical analysis of the compact myelin deposition revealed a progressive decrease in the optical density at 2 and 4 weeks of exposure, followed by significant remyelination at 1 and 2 weeks of recovery (Supplementary Figure S1D,E). A similar temporal pattern was observed for the MBP and MAG, both exhibiting reduced expression during demyelination and gradual restoration during remyelination (Figure 2A–C). These findings indicate a strong association between the dynamic regulation of myelin-associated proteins and the distinct phases of demyelination and remyelination (Figure 2D–F). Immunofluorescence analysis further demonstrated a decrease in mature oligodendrocytes (CC1^+^OLIG2^+^) and oligodendrocyte precursor cells (OPCs, PDGFRα^+^OLIG2^+^) at 2 and 4 weeks of demyelination. Notably, these populations markedly increased at 1 and 2 weeks of remyelination (Figure 2G–J). This dynamic cellular response suggests that OPCs are activated and differentiate into mature oligodendrocytes during remyelination, providing mechanistic insights into myelin repair.

### 3.3. BBB Permeability Dynamics During Cuprizone-Induced Demyelination and Remyelination

**Figure 3:**
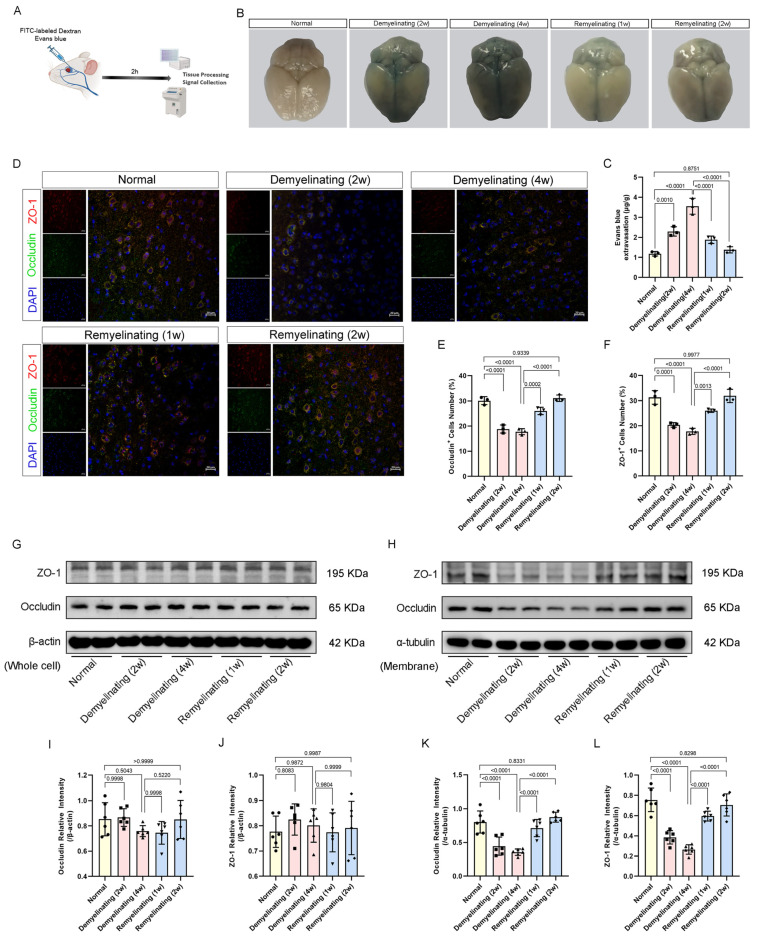
Effects of cuprizone on BBB permeability in mice. (**A**) Schematic of Evans blue and FITC–dextran injection in deeply anesthetized mice at different time points. (**B**) Representative images of EB leakage in brain tissues during demyelination and remyelination phases. (**C**) Quantitative analysis of EB leakage. (**D**) Representative immunofluorescence images of ZO-1 (red) and Occludin (green) in the corpus callosum at different time points during demyelination and remyelination (scale bar: 20 µm). (**E**,**F**) Normalized percentages of ZO-1^+^ and Occludin^+^ cells relative to the total cell population. (**G**) Western blot analysis of ZO-1 and Occludin protein levels in total cell lysates. (**H**) Western blot analysis of ZO-1 and Occludin protein levels in membrane fractions. (**I**,**J**) Quantitative analysis of ZO-1/β-actin and Occludin/β-actin ratios from Western blot images. (**K**,**L**) Quantitative analysis of ZO-1/α-tubulin and Occludin/α-tubulin ratios from Western blot images. Schematic illustrations (**A**) were created via BioRender (https://app.biorender.com/illustrations (accessed on 15 October 2024)). All *p* values were calculated via a one-way ANOVA, followed by Tukey’s multiple comparison test.

Cuprizone-induced demyelination and remyelination represent a dynamic process in which alterations in BBB function may play a critical role. To assess the BBB permeability at different time points, EB dye and FITC-labeled dextran were administered at defined stages (Figure 3A). The EB extravasation increased significantly at 2 and 4 weeks post-cuprizone but was reduced during the first and second weeks of remyelination, indicating BBB restoration (Figure 3B,C). Parallel fluorescence measurements of the FITC–dextran confirmed time-dependent increases in the macromolecule permeability (Supplementary Figure S2A,B).

Immunofluorescence analysis revealed a reduction in ZO1- and Occludin-positive cells at 2 w and 4 w of demyelination, followed by a marked increase during remyelination (1 w and 2 w) (Figure 3D–F). These fluctuations correspond to BBB disruption during demyelination and repair during remyelination. Western blot analysis further revealed that the ZO-1 and Occludin expression in the membrane fraction decreased during demyelination and was restored during remyelination (Figure 3H,K,L). However, the total protein levels of ZO-1 and Occludin did not show significant changes between the groups (Figure 3G,I,J). In addition, the quantitative PCR (qPCR) analysis showed that the transcript levels of TJP1 (encoding ZO-1) and Occludin were not significantly different among the groups (Supplementary Figure S2C,D), suggesting that increased BBB permeability may be attributed to protein mislocalization rather than transcriptional alterations.

### 3.4. A Single-Cell Atlas Revealed Dynamic Cellular Remodeling and Signaling Reprogramming During Demyelination and Remyelination

**Figure 4:**
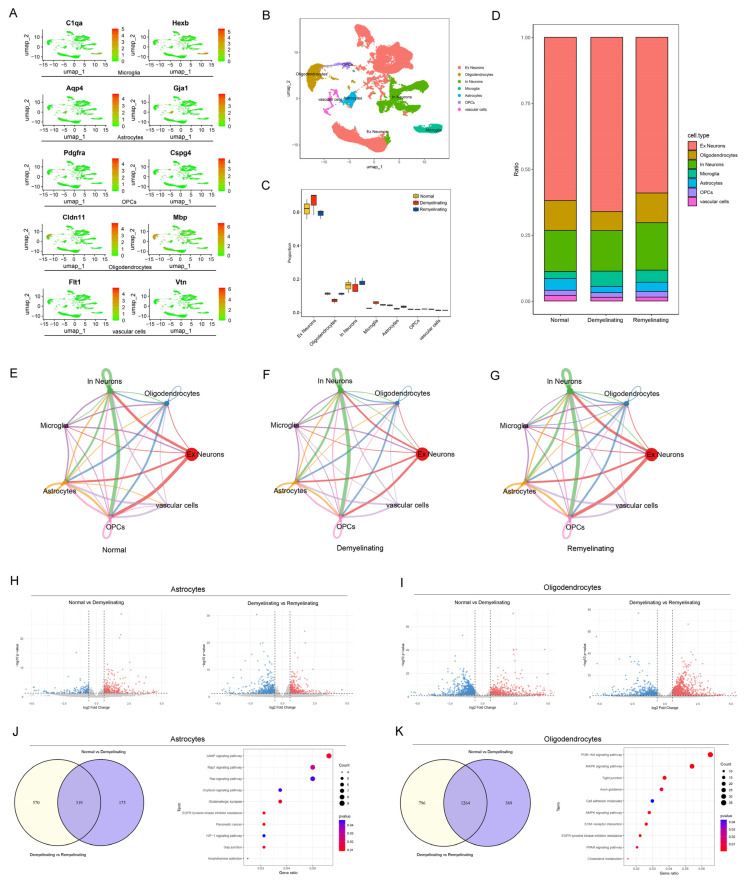
scRNA-seq analysis of the corpus callosum (CC) and cortex. (**A**) Scatter plot showing the distribution of the marker gene expression levels in microglia, astrocytes, OPCs, and oligodendrocytes. (**B**) UMAP plot of the scRNA-seq data. (**C**,**D**) Proportions of each cell type during demyelination and remyelination. (**E**) Receptor–ligand pair counts between different cell populations in normal mice. (**F**) Receptor–ligand pair counts between different cell populations in demyelinated mice. (**G**) Receptor–ligand pair counts between different cell populations in remyelinated mice. (**I**,**J**) Volcano plot displaying DEGs in astrocytes and oligodendrocytes. *p* values were calculated via the Wilcoxon rank-sum test. (**H**) Venn diagram showing overlapping DEGs between normal vs. demyelinated and demyelinated vs. remyelinated astrocytes, along with (**J**) a bar plot of significantly enriched biological pathways for overlapping genes. (**I**) Venn diagram showing overlapping DEGs between OLs (normal vs. demyelinated) and oligodendrocytes (demyelinated vs. remyelinated), along with (**K**) a bar plot of the significantly enriched biological pathways for the overlapping genes.

In a cuprizone-induced mouse model, the demyelination and remyelination processes are closely associated with cellular diversity changes. To systematically dissect the dynamic alterations in glial cells, a comprehensive cell atlas was established by reanalyzing datasets from the GEO database, focusing on the specific heterogeneity in the cortex and corpus callosum (CC) regions. After stringent quality control, 109752 high-quality nuclei were retained. Specific marker genes were used to annotate each cell type. For example, microglia are marked by C1qa and Hexb; astrocytes by Aqp4 and Gja1; OPCs by Pdgfra and Cspg4; oligodendrocytes by Cldn11 and Mbp; vascular cells by Atp13a5 and Cald1; and excitatory and inhibitory neurons by Grin, Syt1, Sv2b, Slc17a7, Gad1, and Gad2 (Figure 4A) (Supplementary Figure S3A). Unsupervised UMAP clustering identified seven transcriptionally distinct neural cell populations: excitatory neurons, inhibitory neurons, OPCs, oligodendrocytes, astrocytes, microglia, and vascular cells (Figure 4B). Further analysis revealed that the cuprizone treatment altered the cell subpopulation proportions, with reductions in the oligodendrocyte, OPC, and astrocyte populations during demyelination and an increase in microglia. These proportions partially recovered after cuprizone withdrawal (Figure 4C,D). The coordinated depletion-recovery dynamics suggest that endogenous regulatory mechanisms maintain cellular diversity despite severe myelin loss.

Cell-cell communication analysis via CellChat in the control, demyelination, and remyelination groups revealed no significant differences in the number or strength of interactions (Figure 4E–G) (Supplementary Figure S3B–D). However, during demyelination, the Psap-Gpr37l1 signaling axis between oligodendrocytes and OPCs was increased, as was the Psap-Gpr37l1 axis from microglia to OPCs, whereas the Psap-Gpr37 axis from astrocytes to oligodendrocytes was reduced (Supplementary Figure S3E–G).

A comparative analysis of the DEGs between the control and demyelination groups, as well as between the demyelination and remyelination groups, revealed changes induced by cuprizone (Figure 4H,I) (Supplementary Figure S3H,I). KEGG pathway analysis further elucidated the biological processes involved in these stages. Astrocytes are involved primarily in the cAMP signaling, Ras signaling, and HIF-1 signaling pathways, which are critical for cell survival and stress responses (Figure 4J). Microglia were enriched in the PI3K-Akt signaling pathway and cytokine-cytokine receptor interaction pathways, highlighting their roles in immune responses and neuroinflammation (Supplementary Figure S3J). Oligodendrocytes were enriched in the PI3K-Akt signaling, MAPK signaling, and axon guidance pathways, indicating their importance in cell proliferation and survival and axonal regeneration (Figure 4K). Finally, OPCs are associated with axon guidance and cell adhesion molecule signaling pathways, further emphasizing their roles in remyelination and cell migration (Supplementary Figure S3K).

### 3.5. Neuroimmune Activation and Oxidative Stress Dynamics During the Demyelination-Remyelination Cycle

**Figure 5:**
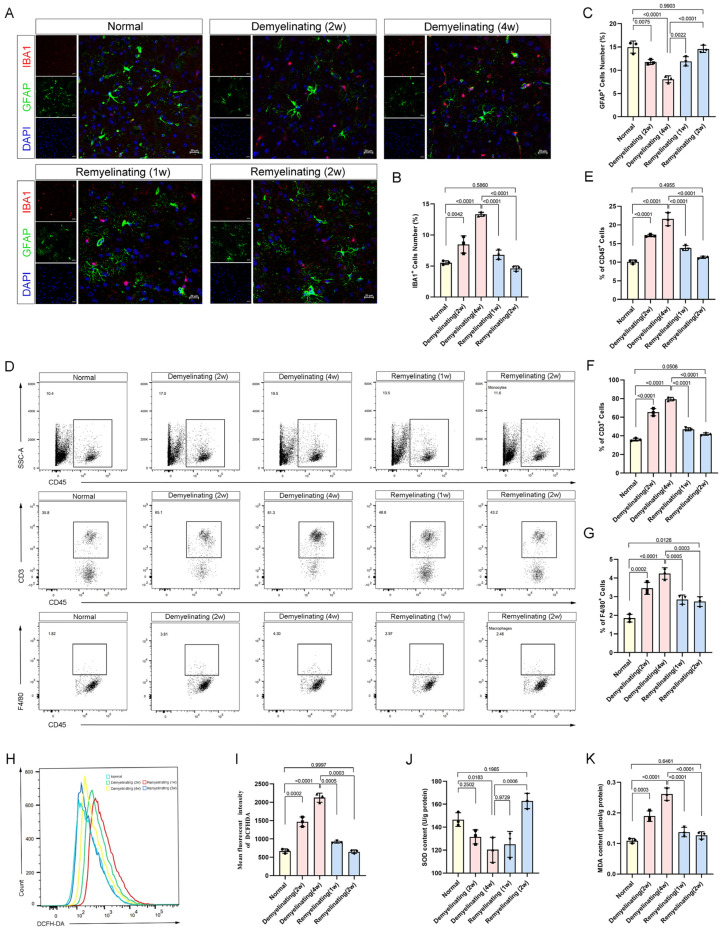
Cuprizone-induced neuroinflammation and immune infiltration. (**A**) Representative immunofluorescence images of IBA1 (red) and GFAP (green) in the cortical region at different time points during demyelination and remyelination (scale bar: 20 µm). (**B**,**C**) Normalized percentages of IBA1+ and GFAP+ cells relative to the total cell population. (**D**) Representative flow cytometry gate strategy for quantifying CD45 (**E**), CD3 (**F**), and F4/80 (**G**) cell populations in the cortex and corpus callosum at different stages of demyelination and remyelination. (**H**,**I**) Flow cytometry analysis of ROS levels in the cortex and corpus callosum. The results of the quantitative analysis of the ROS generation are displayed as bar graphs. (**J**,**K**) Kit-based detection of SOD and MDA activity in the cortex and corpus callosum. All *p* values were calculated via a one-way ANOVA, followed by Tukey’s multiple comparison test.

Cuprizone-induced demyelination and subsequent remyelination are accompanied by significant alterations in the BBB integrity and cellular heterogeneity. These processes are likely influenced by neuroimmune responses and oxidative stress, which play pivotal roles in driving disease pathology. The dual immunolabeling of microglia and astrocytes revealed a time-dependent increase in the IBA1^+^ microglial density during demyelination, whereas the GFAP^+^ astrocytic reactivity was significantly suppressed, indicating functional divergence in the glial responses (Figure 5A–C). This trend reversed during remyelination, suggesting a temporal coordinated transition from microglia-driven inflammation to astrocyte-mediated repair (Figure 5A–C).

Flow cytometry further revealed significant shifts in the immune cell populations within the cortex and corpus callosum (Figure 4D). At weeks 2 and 4 of demyelination, the proportions of CD45^+^ immune cells, CD3^+^ T cells, and F4/80^+^ macrophages markedly increased, indicating a sustained immune response (Figure 5E–G). During remyelination, the proportions of these immune cells decreased significantly at weeks 1 and 2, suggesting the resolution of inflammation and the initiation of tissue repair (Figure 4E–G).

Oxidative stress markers further highlighted cuprizone’s impact on cellular damage. At weeks 2 and 4 of demyelination, the reactive oxygen species (ROS) and malondialdehyde (MDA) levels were significantly elevated, whereas the superoxide dismutase (SOD) activity was decreased, reflecting increased oxidative stress and exacerbating cellular injury (Figure 5H–K). Consistently, the qPCR analysis revealed that the SOD1 transcription was downregulated during demyelination and subsequently restored during the remyelination phase, further supporting the involvement of antioxidant gene regulation in the recovery process (Supplementary Figure S4A). Upon cuprizone withdrawal, these oxidative stress markers improved during the first two weeks of remyelination (Figure 5H–K), indicating the reactivation of endogenous antioxidant defense mechanisms during the repair process.

### 3.6. Transcriptomic Analysis and Inflammatory Regulatory Networks in Cuprizone-Induced Demyelination and Remyelination

**Figure 6:**
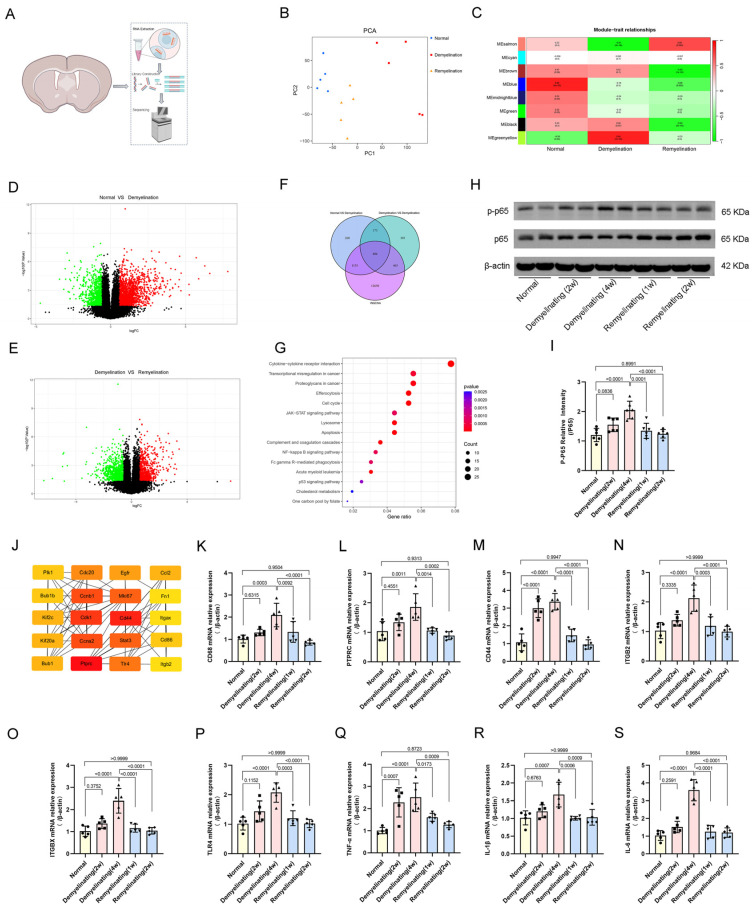
Bioinformatics analysis of transcriptomes in the cortex and corpus callosum. (**A**) Schematic of the experimental design for transcriptome sequencing. (**B**) Principal component analysis (PCA) plot illustrating the variability among biological replicates. (**C**) WGCNA identifies gene modules associated with demyelination and remyelination. The heatmap displays correlations and *p* values between modules and traits. (**D**) The volcano plot shows differentially expressed genes (DEGs) between the normal and deceased groups. *p* values were calculated via the Wilcoxon rank-sum test. (**E**) Volcano plot showing DEGs between the demyelinated and remyelinated groups. *p* values were calculated via the Wilcoxon rank-sum test. (**F**,**G**) Venn diagram illustrating overlapping DEGs between normal and demyelinated groups and demyelinated and remyelinated groups, with bar plots displaying significantly enriched biological pathways for overlapping genes. (**H**) Western blot analysis of p65 and p-p65 protein levels in total cell lysates. (**I**) Quantitative analysis of p65/p-p65 from Western blot images. (**J**) A protein-protein interaction (PPI) network was constructed via the STRING database, with hub genes identified via Cytoscape. (**K**–**P**) qPCR validation of the mRNA expression levels of inflammation-related hub genes normalized to those of β-actin. (**Q**–**S**) qPCR validation of inflammatory cytokine mRNA expression levels normalized to those of β-actin. Schematic illustrations (**A**) were created via BioRender. All *p* values were calculated via a one-way ANOVA, followed by Tukey’s multiple comparison test.

The critical role of neuroinflammation in cuprizone-induced demyelination and subsequent remyelination underscores the molecular mechanisms governing these processes. The bulk RNA sequencing of cortical and corpus callosum tissues systematically revealed dynamic transcriptional reprogramming associated with cuprizone-induced demyelination and repair (Figure 6A). Principal component analysis revealed a distinct separation in the gene expression profiles between the demyelination group and both the control and remyelination groups, highlighting the extensive transcriptional alterations following myelin loss (Figure 6B). Weighted gene coexpression network analysis, with the soft threshold set at 10, identified eight distinct expression modules (Supplementary Figure S3B,C). Clustering analysis and correlation heatmaps revealed a significant negative correlation between demyelination and MEsalmon, whereas remyelination was inversely associated with MEbrown, providing insights into module-specific functions during pathological damage and repair (Figure 6C). Differential gene expression analysis between the normal and demyelination groups, as well as between the demyelination and remyelination groups, confirmed a marked shift in transcriptional regulation following cuprizone exposure (Figure 6D–E). A KEGG pathway mapping of the overlapping DEGs revealed cytokine-receptor interactions, JAK-STAT signaling, and NF-κB signaling as key regulators of these transcriptional changes (Figure 6F-G). Western blot analysis further demonstrated that the phosphorylation level of the NF-κB pathway component p65 was markedly increased during the demyelination phase and decreased during the remyelination phase (Figure 2H–I), supporting the key role of NF-κB signaling in neuroinflammation dynamic regulation.

Further protein-protein interaction analyses via the STRING database (Supplementary Figure S3D) revealed 20 hub genes (Figure 6J), whose dynamic transcriptional patterns in the cortex and corpus callosum were visualized via heatmaps (Supplementary Figure S3E). The functional annotation suggested strong associations between these core genes and tumorigenesis, cardiovascular dysfunction, apoptosis regulation, endocrine–metabolic disorders, and immune-related diseases (Supplementary Figure S3F). Finally, the qPCR validation revealed the significant upregulation of inflammation-associated hub genes and key inflammatory mediators, including IL-6, IL-1β, and TNF-α, during demyelination, with a marked decline during remyelination (Figure 6K–S). These findings not only validate the transcriptomic analyses but also highlight the dynamic regulation of inflammatory signaling as a potential bidirectional modulator of myelin injury and repair.

## 4. Discussion

The primary pathological hallmark of demyelinated diseases is the widespread loss of myelin, leading to reduced nerve conduction efficiency and subsequent sensory-motor and cognitive dysfunction [27,28,29]. Compared with other models, the oral cuprizone administration model offers advantages in terms of its rapid induction, reproducibility, and minimal trauma [30,31]. This study demonstrated that cuprizone-induced demyelination progressively impairs sensory–motor and cognitive functions, whereas remyelination partially reverses these deficits. The sensory-motor impairments observed at two weeks post-demyelination correlate with a decrease in the axonal conduction efficiency and neuronal hyperexcitability, which is consistent with previous findings [32,33] on delayed action potential propagation in the motor cortex following demyelination. Behavioral recovery temporal dynamics reveal critical functional repair windows. Improvements in sensory-motor functions during remyelination are synchronized with OPC differentiation and de novo myelin formation, a process that can be accelerated by activity-dependent mechanisms [34]. The limited recovery of cognitive function in the novel object recognition task reflects the partial restoration of the myelin integrity, which aligns with mechanisms by which adaptive myelination supports synaptic plasticity and memory consolidation [35,36].

The dynamic interplay between demyelination and endogenous remyelination constitutes a critical yet incompletely understood axis in CNS repair mechanisms. This study elucidates the spatiotemporal patterns of oligodendrocyte reconstruction during cuprizone-induced myelin remodeling, revealing stage-dependent structural and cellular adaptive changes. Quantitative LFB staining revealed progressive demyelination during weeks 2–4 of cuprizone exposure, followed by significant remyelination during weeks 1–2 of recovery, a trajectory paralleled by fluctuations in the MBP/MAG protein expression. The temporal alignment of the myelin structural and molecular changes highlights myelin plasticity during degeneration and regeneration. The number of oligodendrocyte precursor cells (OPCs, PDGFRα^+^OLIG2^+^) and mature oligodendrocytes (CC1^+^OLIG2^+^) decreased during demyelination but increased significantly during remyelination, indicating that OPC activation and differentiation are central drivers of endogenous repair. This cellular plasticity resembles the differentiation cascade observed in zebrafish CNS regeneration models, where radial glial cells transiently acquire OPC-like properties to facilitate repair [37]. Additionally, the activation of endogenous neural stem cells (NSCs) in multiple sclerosis to replenish the oligodendrocyte lineage further supports OPC mobilization as a conserved repair pathway [38,39].

The BBB serves as a critical interface to regulate central nervous system homeostasis, and its dysfunction is closely associated with demyelinating diseases such as multiple sclerosis [40,41]. BBB permeability disruption may exacerbate neuroinflammatory cascades and hinder myelin regeneration [42,43]. This study demonstrated that dynamic changes in the BBB permeability are linked to cuprizone-induced demyelination and subsequent myelin regeneration. Both small (EB) and large (FITC–dextran) molecular tracer extravasations exhibited synchronous changes, suggesting compromised endothelial junction stability rather than irreversible structural damage. A transient reduction in the membrane localization of ZO-1 and Occludin (with unchanged total protein levels) indicates that posttranslational redistribution or cytoskeletal disruption during the demyelination phase may lead to decreased junction stability. Further supporting this notion, the qPCR analysis showed no significant changes in the mRNA expression of TJP1 and Occludin, suggesting that the observed junctional defects are likely resulting from intracellular trafficking alterations, protein anchorage failure, or interaction with inflammatory signaling pathways rather than transcriptional regulation. In the regeneration phase, these proteins may be reorganized by anti-inflammatory signals or OPC recruitment [44,45,46]. The restoration of the BBB integrity occurs in parallel with the reorganization of tight junction structures, which may provide a favorable microenvironment for OPC recruitment and differentiation. During the demyelination phase, microglia exacerbate the damage by releasing proinflammatory cytokines (e.g., TNF-α and IL-1β) [47,48], whereas in the regeneration phase, they switch to a phagocytic and anti-inflammatory phenotype that aids in myelin debris clearance and neurotrophic factor secretion [49]. For example, Gharibani et al. report that kinase C modulators could reduce microglial activation and accelerate myelin regeneration, validating the interplay between neuroinflammation and barrier repair [50]. Transient BBB disruption during demyelination may allow peripheral immune mediators to infiltrate, thereby exacerbating inflammation and oligodendrocyte damage, whereas barrier repair during regeneration may limit further injury and promote local repair signaling.

scRNA-seq has played a pivotal role in elucidating the cellular heterogeneity and dynamic remodeling mechanisms underlying neurodegenerative processes [51,52]. In the cuprizone-induced demyelination and remyelination model, significant changes in the cellular composition and signaling dynamics were observed. During the demyelination phase, the proportion of oligodendrocytes and astrocytes decreased, whereas the proportion of microglia increased, indicating neuroinflammation activation. In the remyelination phase, these proportions partially recovered, reflecting the activation of endogenous repair mechanisms. An analysis of the intercellular signaling revealed that during demyelination, the Psap-Gpr37l1 signaling axis between oligodendrocytes and OPCs was upregulated, and microglia also presented increased Psap-Gpr37l1 signaling to OPCs. Conversely, astrocytes exhibited a reduction in the Psap-Gpr37 signaling axis with oligodendrocytes. These signaling alterations may play crucial roles in myelin injury and repair [53,54]. KEGG pathway analysis revealed that astrocytes specifically activate the cAMP and HIF-1 pathways to respond to metabolic stress, which is consistent with their core role in maintaining redox homeostasis [55,56]. Microglia are enriched in the cytokine-receptor interaction pathway, which aligns with the transcriptional signature of disease-associated microglia in neurodegenerative disorders [57]. The coactivation of the PI3K-Akt and MAPK pathways in oligodendrocyte lineage cells reflects their classical role in regulating the survival-differentiation balance during myelin repair [58,59,60]. Compared with the original study on GSE204769, which focused on defining DOL and MAFB^hi^ microglia [26], this study systematically quantified regional changes in the cellular composition and transcriptional features and integrated intercellular communication networks. These analyses further revealed a potential role for the Psap–Gpr37l1 signaling axis in regulating glial interactions. Future experiments may consider using the conditional knockout or pharmacological blockade of the Psap–Gpr37l1 pathway to verify its functional necessity in OPC replenishment and blood–brain barrier repair, offering translational insights into demyelinating diseases.

Neuroinflammation plays a pivotal role in neurodegenerative disease onset and progression. Microglia and astrocyte activation is a hallmark of neuroinflammation [61,62], with excessive inflammatory responses potentially leading to neuronal damage and disease progression. This study reveals the spatiotemporal dynamics of neuroimmune activation and oxidative stress during the cuprizone-induced demyelination-remyelination cycle. The inverse dynamics of microglial proliferation and astrocytic quiescence during acute demyelination, along with their functional reverse during the repair phase, suggests the existence of a “functional division” paradigm in glial cell pathological responses [14]. This biphasic regulation correlates with the phenotypic switch in astrocytes—when inflammation subsides, the proinflammatory A1 phenotype converts to a neuroprotective A2 state [63], whereas microglia shift from phagocytic activation to immune regulatory functions [64,65]. The persistent elevation of CD45^+^ leukocytes and tissue-resident macrophages during the demyelination phase, coupled with progressive oxidative damage marked by ROS/MDA accumulation and SOD depletion, confirms that chronic neuroinflammation is both a consequence and a driver of myelin toxicity. The transcriptomic analysis further revealed the global reprogramming of inflammatory and stress pathways. Specifically, Tlr4-mediated NF-κB activation drives the upregulation of proinflammatory cytokines (IL6, IL1β, and TNFα), which is consistent with its role in amplifying microglial reactivity in experimental autoimmune encephalomyelitis [65,66]. Conversely, the downregulation of *Itgax* (CD11c) during the remyelination phase may reflect reduced antigen-presenting functions, suggesting the attenuation of immune surveillance during the repair phase. The sustained high expression of *Ptprc* (CD45) in demyelinated tissue is correlated with T-cell infiltration in human demyelinating plaques [67], whereas the downregulation of *Itgb2* may reduce leukocyte adhesion [68,69]. These hub genes collectively form a molecular scaffold linking neuroinflammation with metabolic apoptosis pathways and may regulate oligodendrocyte precursor cell survival.

**Figure 7:**
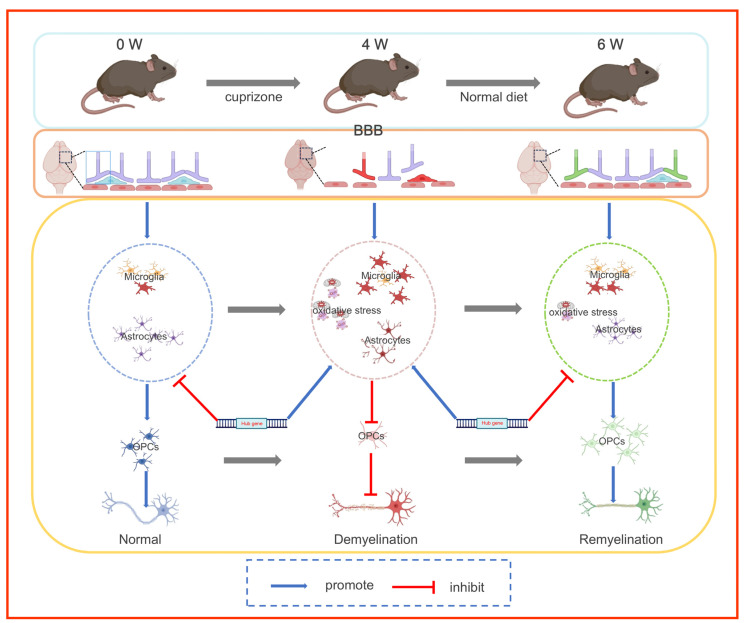
Schematic overview of cuprizone-induced demyelination and remyelination mechanisms.

In summary, the cuprizone-induced demyelination model reveals a dynamic relationship between endogenous myelin regeneration and functional recovery (Figure 7). Sensorimotor and cognitive deficits correlate temporally with myelin loss, whereas the regeneration process partially reverses neurological dysfunction. Oligodendrocyte population reconstruction, particularly precursor cell differentiation, aligns with BBB integrity restoration and neuroinflammation resolution. Single-cell profiling revealed a coordinated cellular remodeling process, whereas transcriptomic analysis emphasized the dominance of inflammatory regulatory networks in driving the demyelination-repair cycle. The simultaneous alleviation of oxidative stress and immune infiltration during the remyelination phase further highlights the critical role of the microenvironment in regulating repair. These findings suggest that modulating neuroimmune interactions and enhancing endogenous repair mechanisms may provide novel therapeutic strategies for treating demyelinating diseases.

## Data Availability

The authors declare that all supporting data and materials are available within the article, and the raw data can be obtained under reasonable requirements from the corresponding author.

## Supplementary Materials

The following supporting information can be downloaded at https://www.mdpi.com/article/10.3390/antiox14060692/s1, Figure S1: Representative gating strategy and alterations in sensorimotor function during demyelination and remyelination in cuprizone-induced model mice. (A) Gating strategy for flow cytometry-based quantification of CD45, CD3, and F4/80 cell populations infiltrating the cerebral cortex and corpus callosum. (B) Time required for mice to contact (left panel) and remove (right panel) adhesive tape placed on their forepaws. (C) Assessment of foot fault rates in mice. (D) Representative images of Luxol fast blue (LFB) staining of the corpus callosum at different time points. (E) Quantitative analysis of the integrated optical density (IOD) for LFB staining. All *p* values were calculated via a one-way ANOVA, followed by Tukey’s multiple comparison test.; Figure S2: (A) Representative images of FITC-dextran extravasation in the cerebral cortex at different time points (scale bar: 1 mm). (B) Quantitative analysis of the FITC-dextran extravasation area. (C-D) The mRNA expression levels of TJP1 and Occludin were validated by qPCR and normalized against β-actin. All *p* values were calculated via one-way ANOVA, followed by Tukey’s multiple comparison test. Figure S3: Supplementary analysis of single-nucleus RNA sequencing data from the corpus callosum and cortex. (A) Expression levels of marker genes in seven cell populations. (B) Receptor-ligand interaction strengths between different cell populations in the normal mouse group. (C) Receptor-ligand interaction strengths between different cell populations in the demyelinated mouse group. (D) Receptor-ligand interaction strengths between different cell populations in the remyelinated mouse group. (E-F) Distribution of 14 receptor-ligand pairs in different cell populations. (H-I) Volcano plots illustrating differentially expressed genes (DEGs) in microglia and OPCs. *p* values were calculated via the Wilcoxon rank-sum test. (J) Venn diagram showing overlapping DEGs between the Normal vs Demyelinated groups and between the Demyelinated vs Remyelinated groups in astrocytes, along with a bar plot of significantly enriched biological pathways for the overlapping genes in microglia. (K) Venn diagram showing overlapping DEGs between the Normal vs Demyelinated and Demyelinated vs Remyelinated groups in OPCs, along with a bar plot of significantly enriched biological pathways for the overlapping genes in OPCs. Figure S4: Supplementary analysis of transcriptomes in the cortex and corpus callosum. (A) SOD1 mRNA expression was validated by qPCR and normalized against β-actin. All *p* values were calculated via one-way ANOVA, followed by Tukey’s multiple comparison test. (B) Identification of gene modules associated with demyelination and remyelination via WGCNA. The “soft” threshold was selected based on a comprehensive analysis of scale independence and mean connectivity. (C) Different colors represent gene coexpression modules on the gene dendrogram. (D) Construction of a protein-protein interaction (PPI) network for overlapping genes via String. (E) Heatmap showing transcriptional changes in the top 20 hub genes. (F) Alluvial plot illustrating the biological pathways significantly enriched by each hub gene. Table S1: The Primer Sequences for qRT-PCR Analysis.
