# Supplementary material for: Dynamic Transcriptomic and Cellular Remodeling Underlie Cuprizone-Induced Demyelination and Endogenous Repair in the CNS
Source: Antioxidants (Basel). 2025 Jun 6;14(6):692. doi: 10.3390/antiox14060692 (PMC12189453; doi:10.3390/antiox14060692)
Supplement: Supplementary file 1 [file antioxidants-14-00692-s001.zip › Supplemental Materials.pdf]

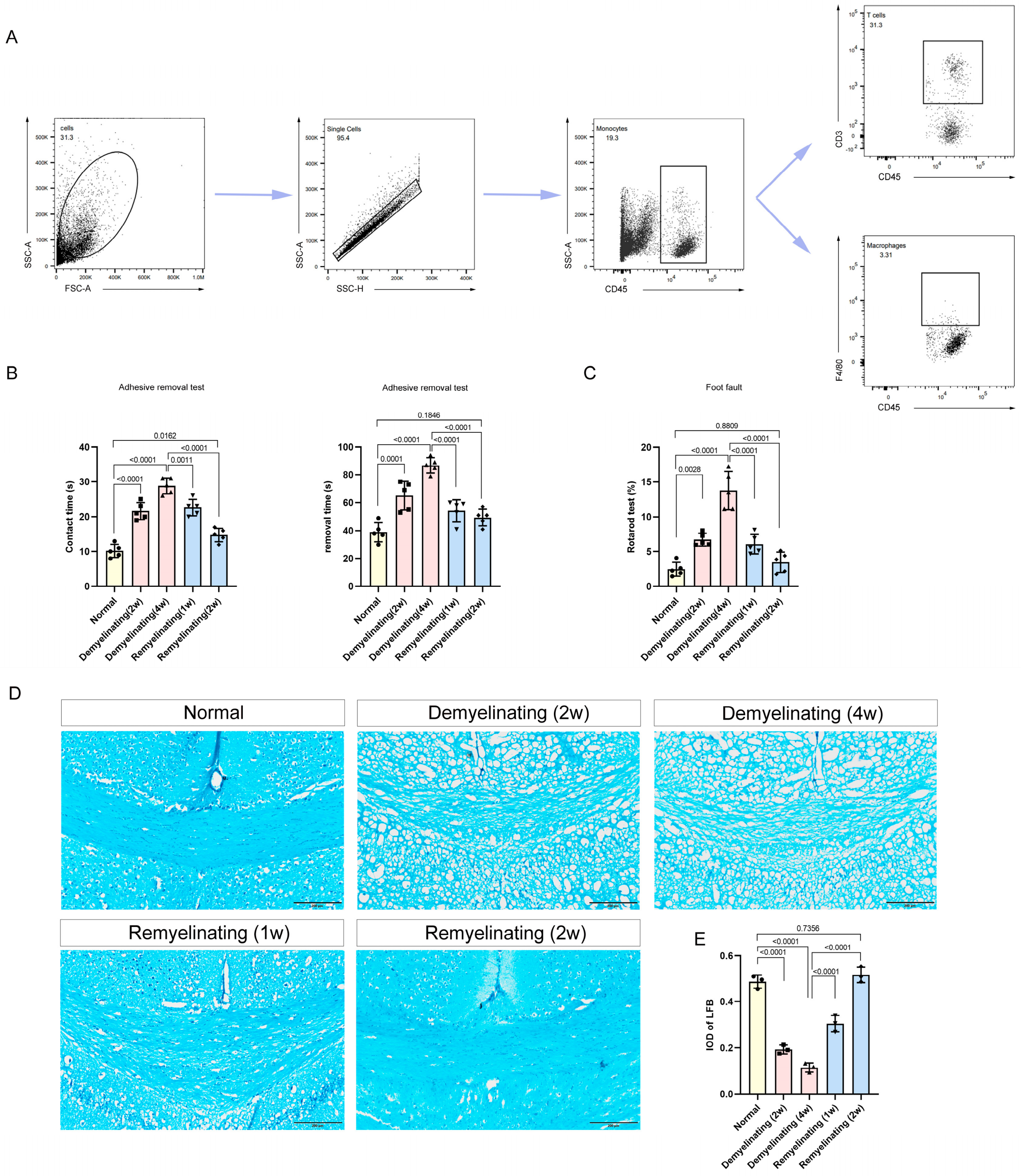

Supplementary Figure S1 Representative gating strategy and alterations in sensorimotor function during demyelination and remyelination in cuprizone-induced model mice. (A) Gating strategy for flow cytometry-based quantification of CD45, CD3, and F4/80 cell populations infiltrating the cerebral cortex and corpus callosum. (B) Time required for mice to contact (left panel) and remove (right panel) adhesive tape placed on their forepaws. (C) Assessment of foot fault rates in mice. (D) Representative images of Luxol fast blue (LFB) staining of the corpus callosum at different time points. (E) Quantitative analysis of the integrated optical density (IOD) for LFB staining. All p values were calculated via a one-way ANOVA, followed by Tukey's multiple comparison test.

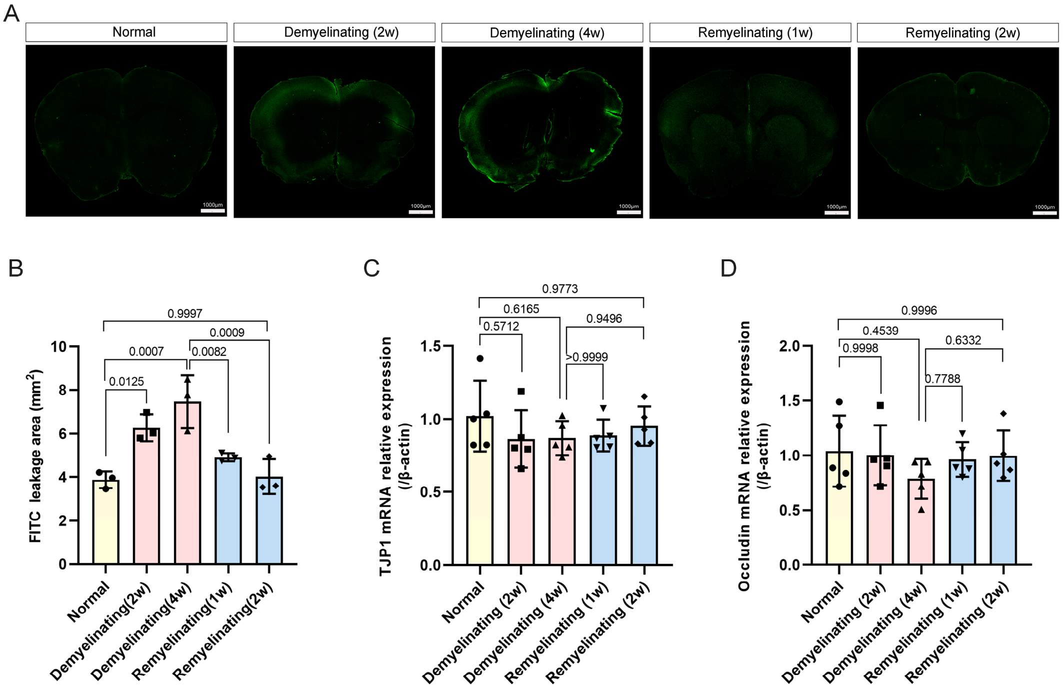

Supplementary Figure S2 (A) Representative images of FITC-dextran extravasation in the cerebral cortex at different time points (scale bar: 1 mm). (B) Quantitative analysis of the FITC-dextran extravasation area. (C-D) The mRNA expression levels of TJP1 and Occludin were validated by qPCR and normalized against β-actin. All p values were calculated via one-way ANOVA, followed by Tukey's multiple comparison test.

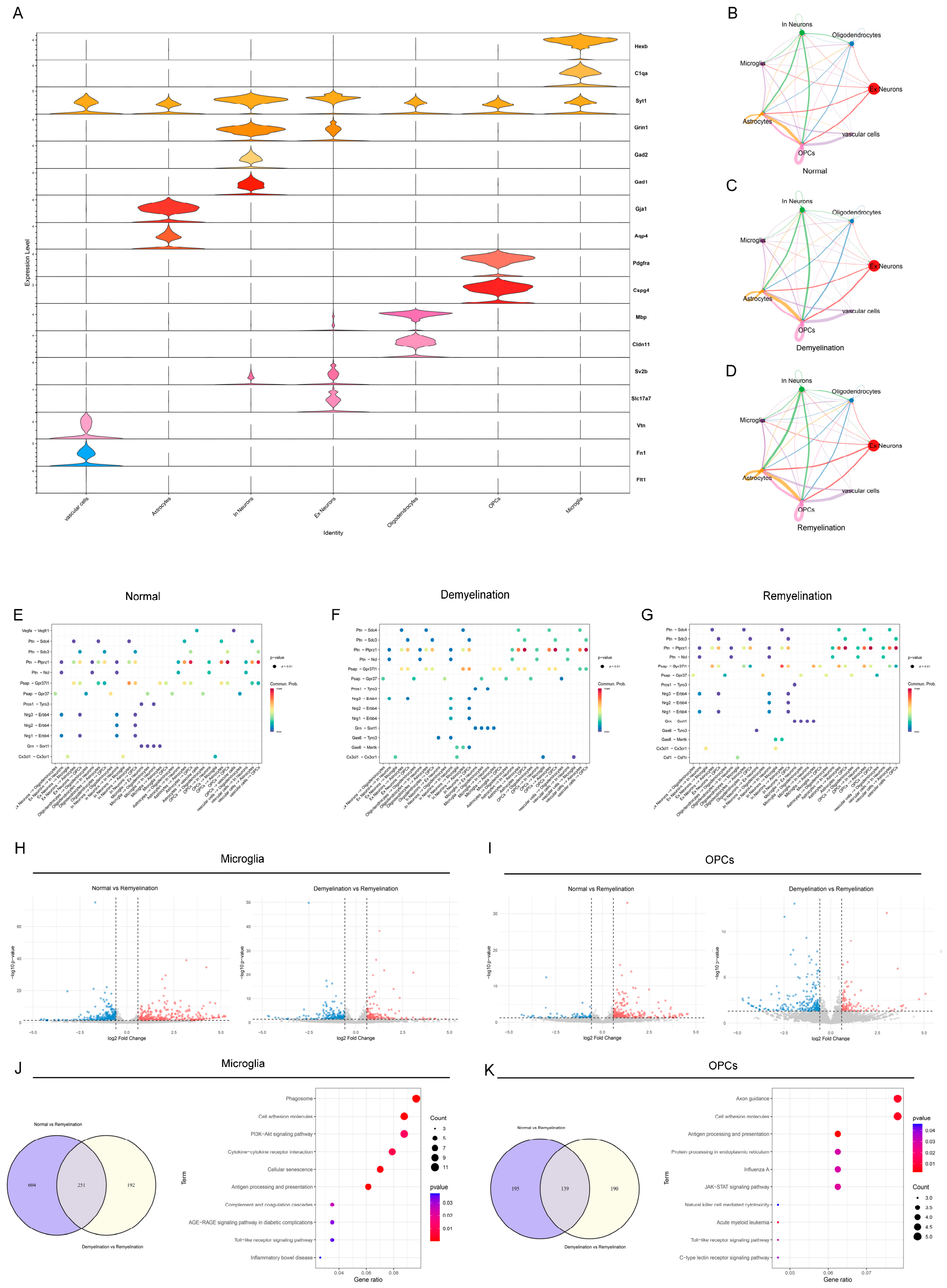

Supplementary Figure S3 Supplementary analysis of single-nucleus RNA sequencing data from the corpus callosum and cortex. (A) Expression levels of marker genes in seven cell populations. (B) Receptor–ligand interaction strengths between different cell populations in the normal mouse group. (C) Receptor–ligand interaction strengths between different cell populations in the demyelinated mouse group. (D) Receptor-ligand interaction strengths between different cell populations in the remyelinated mouse group. (E-F) Distribution of 14 receptor–ligand pairs in

different cell populations. (H-I) Volcano plots illustrating differentially expressed genes (DEGs) in microglia and OPCs. p values were calculated via the Wilcoxon rank-sum test. (J) Venn diagram showing overlapping DEGs between the Normal vs Demyelinated groups and between the Demyelinated vs Remyelinated groups in astrocytes, along with a bar plot of significantly enriched biological pathways for the overlapping genes in microglia. (K) Venn diagram showing overlapping DEGs between the Normal vs Demyelinated and Demyelinated vs Remyelinated groups in OPCs, along with a bar plot of significantly enriched biological pathways for the overlapping genes in OPCs.

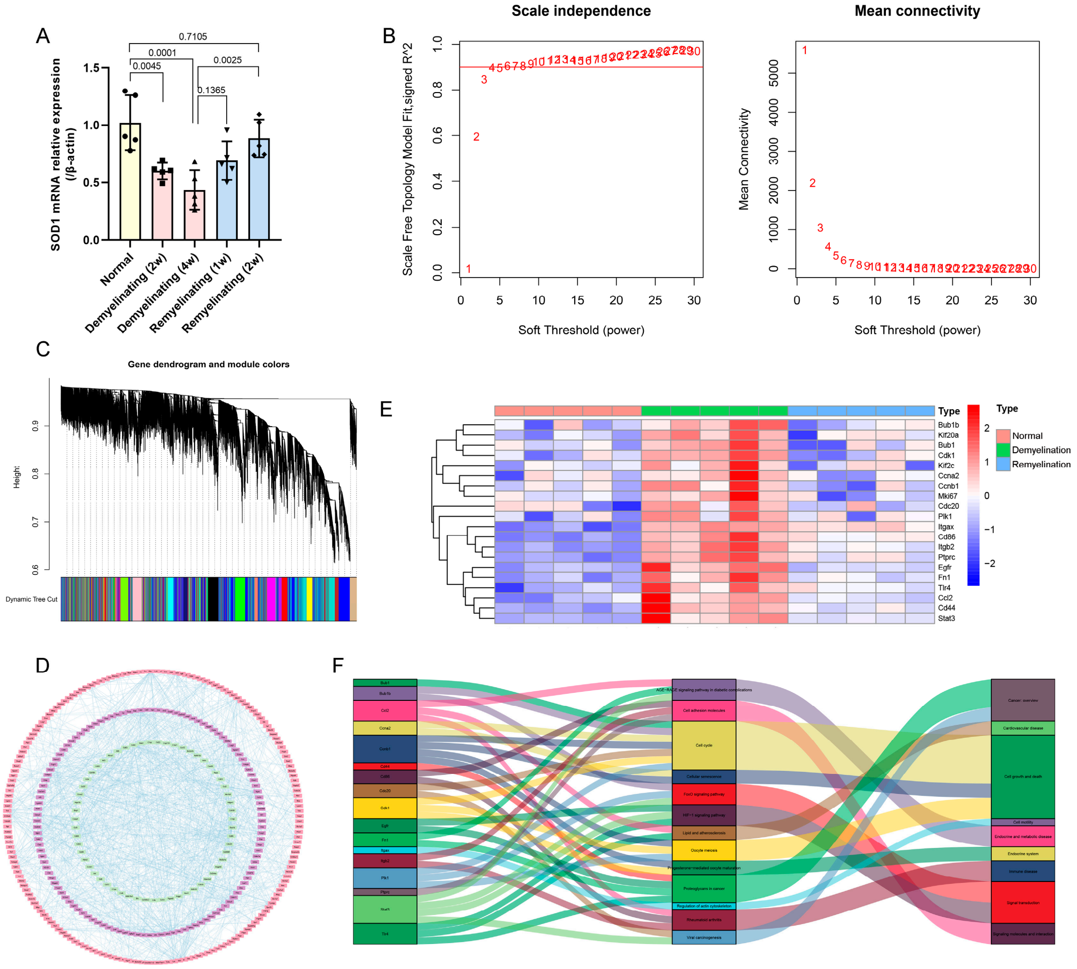

Supplementary Figure S4 Supplementary analysis of transcriptomes in the cortex and corpus callosum. (A) SOD1 mRNA expression was validated by qPCR and normalized against β-actin. All p values were calculated via one-way ANOVA, followed by Tukey's multiple comparison test. (B) Identification of gene modules associated with demyelination and remyelination via WGCNA. The "soft" threshold was selected based on a comprehensive analysis of scale independence and mean connectivity. (C) Different colors represent gene coexpression modules on the gene dendrogram. (D) Construction of a protein–protein interaction (PPI) network for overlapping genes via String. (E) Heatmap showing transcriptional changes in the top 20 hub genes. (F) Alluvial plot illustrating the biological pathways significantly enriched by each hub gene.
