# Supplementary material for: Dynamic Transcriptomic and Cellular Remodeling Underlie Cuprizone-Induced Demyelination and Endogenous Repair in the CNS
Source: Antioxidants (Basel). 2025 Jun 6;14(6):692. doi: 10.3390/antiox14060692 (PMC12189453; doi:10.3390/antiox14060692)
Supplement: Supplementary file 1 [file antioxidants-14-00692-s001.zip › Supplementary Table S1.pdf]

The Primer Sequences for qRT-PCR Analysis

Genes	Sense	Antisense
Ptprc	GTGCATGTAGCTAGCAAGTGG	GGTGTAGGTGTTTGCCCTGT
Cd44	CACCTTGGCCACCACTCCTAAT	CCCTTCTGTCACATGGGAGTC
Tlr4	TTGAATCCCTGCATAGAGGTAG	TTCAAGGGGTTGAAGCTCAGAT
Itgb2	CCAAGAGGTTCCACCCACAT	AGACTCCACAAAGCTTACCTTC
Itgax	GACCTAGCACACGGTTCTCC	GAGCTCCACTTTGGGTGGTG
Cd86	CAGCACGGACTTGAACAACC	CTCCACGGAAACAGCATCTGA
TNF-α	CCCTCACACTCACAAACCAC	ACAAGGTACAACCCATCGGC
IL-1β	TGCCACCTTTTGACAGTGATG	AAGGTCCACGGGAAAGACAC
IL-6	GACAAAGCCAGAGTCCTTCAGA	GTGACTCCAGCTTATCTCTTGGT
TJP1	GGAGATGTTTATGCGGACGGT	CTCCATTGCTGTGCTCTTAGC
Occludin	CCCTCTTTCCTTAGGCGACA	CCCAAGATAAGCGAACCTGC
SOD1	CATGGCGATGAAAGCGGTG	GCACTGGTACAGCCTTGTGTA
β-actin	ACTGTCGAGTCGCGTCCA	ATCCATGGCGAACTGGTGG
